# Molecular mechanisms regulating formation, trafficking and processing of annular gap junctions

**DOI:** 10.1186/s12860-016-0087-7

**Published:** 2016-05-24

**Authors:** Matthias M. Falk, Cheryl L. Bell, Rachael M. Kells Andrews, Sandra A. Murray

**Affiliations:** Department of Biological Sciences, Lehigh University, Bethlehem, PA 18049 USA; Department of Cell Biology and Physiology, University of Pittsburgh, School of Medicine, Pittsburgh, PA l5261 USA

**Keywords:** Annular, Clathrin, Connexin, Degradation, Dynamin, Endocytosis, Fission, Gap Junction, Lysosomes, Phosphorylation, Ubiquitination, ZO-1

## Abstract

Internalization of gap junction plaques results in the formation of annular gap junction vesicles. The factors that regulate the coordinated internalization of the gap junction plaques to form annular gap junction vesicles, and the subsequent events involved in annular gap junction processing have only relatively recently been investigated in detail. However it is becoming clear that while annular gap junction vesicles have been demonstrated to be degraded by autophagosomal and endo-lysosomal pathways, they undergo a number of additional processing events. Here, we characterize the morphology of the annular gap junction vesicle and review the current knowledge of the processes involved in their formation, fission, fusion, and degradation. In addition, we address the possibility for connexin protein recycling back to the plasma membrane to contribute to gap junction formation and intercellular communication. Information on gap junction plaque removal from the plasma membrane and the subsequent processing of annular gap junction vesicles is critical to our understanding of cell-cell communication as it relates to events regulating development, cell homeostasis, unstable proliferation of cancer cells, wound healing, changes in the ischemic heart, and many other physiological and pathological cellular phenomena.

## Background

**Fig. 1:**
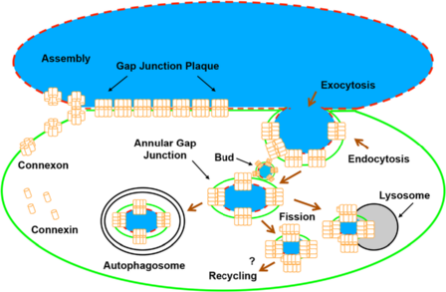
Schematic diagram illustrating the formation of an annular gap junction from a gap junction plaque and its subsequent processing. Newly synthesized connexin (Cx) proteins assemble into a six-protein oligomer called a connexon. Connexons are then transported to and inserted into the plasma membrane. During cell-cell contact, a hemichannel can dock head-on with a hemichannel from an apposing cell and cluster to form a gap junction plaque. Once the plaque is no longer needed for cell-cell communication, or a cell becomes migratory, a portion of the plaque (usually the central portion) or the entire plaque is internalized to form an annular gap junction. The annular gap junction may then be degraded via a number of processes, including endo-lysosomal, autophagosomal, or a fission process followed by lysosomal degradation (as shown). It has been suggested that annular gap junction vesicles or free connexin proteins in cytoplasmic membranes that are released during annular gap junction vesicle degradation may recycle back to the cell surface to participate in the formation of new, or the addition to existing gap junction plaques. Black arrows depict structural components, while maroon arrows depict cellular processes

Gap junctions are membrane channels composed of proteins termed connexins [1, 2]. These channels permit intercellular communication of regulatory molecules that are thought to play a pivotal role in regulating a vast number of normal and diseased cellular events, including those during development, differentiation, and functions of most cells of the body [3–5]. Many cells express more than one of the twenty members of the connexin family that have now been identified in humans [1, 2, 6] and channels composed of more than one connexin type have been reported [1]. Gap junction channels generally form between cells of the same type, but they can also form between cells of different types [7, 8]. Connexin 43 (Cx43) gap junction protein, the most ubiquitously expressed connexin, has been shown to be synthesized in the endoplasmic reticulum (ER), oligomerized into a hemichannel in the Golgi and then transported to the cell surface [6, 9–11]. On the cell surface, hemichannels (termed connexons) from apposing cells align and dock head-on into complete, double-membrane spanning gap junction channels [12]. These channels then aggregate into gap junction plaques [1]. Although an isolated gap junction hemichannel within the cell membrane may be functional [13, 14], most channels are thought only to be functional once within gap junction plaques [1, 15]. The availability of functional channels between apposing cells is needed for efficient cell-cell communication, and therefore the removal of gap junction channels from the cell surface critically impacts the capacity for communication. It is widely accepted that gap junction channel removal from the cell surface involves a distinctive gap junction plaque internalization process, which results in the formation of annular gap junction vesicles in the cytoplasm of one of two contacting cells (Fig. 1). Processing of annular gap junction vesicles for degradation by autophagosomal and endo-lysosomal pathways has been described [16–23]. A potential return of annular gap junction connexins back to the cell surface, a provocative yet appealing thought, has recently been given some attention as well. The events in the annular gap junction “life cycle” have been suggested to regulate the number of gap junction channels available for communication and thus cell physiological functions [24–28].

In this article, we will first discuss the morphological characteristics of annular gap junction vesicles as revealed over time with combined multi-morphological imaging techniques. Then, we will review the current knowledge on annular gap junction formation and subsequent processing including fission, degradation and potential connexin protein recycling. We also discuss the known cellular proteins that regulate these processing events.

## Characterization of gap junction and annular gap junction structures

### Transmission Electron Microscopy (TEM)

**Fig. 2:**
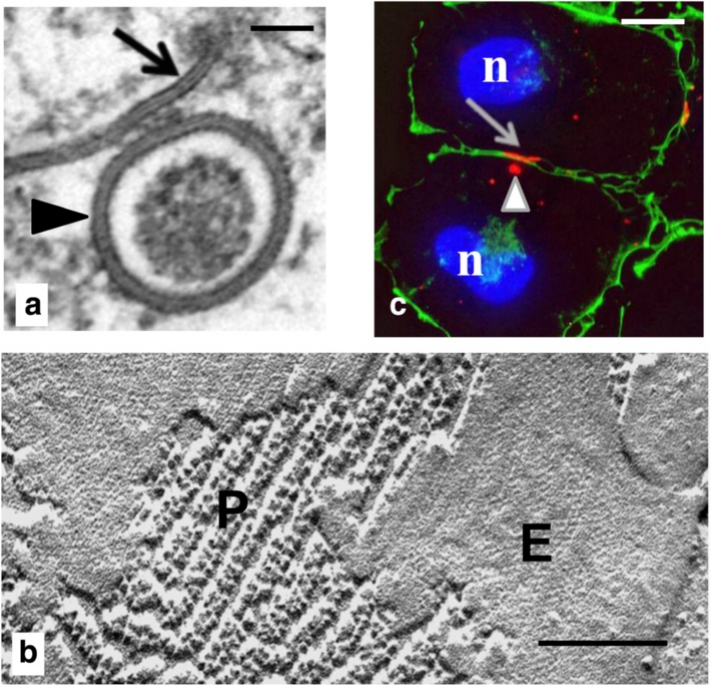
Gap junction plaques (*arrows*) and annular gap junction vesicles (*arrowheads*) shown with transmission electron (**a**), freeze fracture electron (**b**), and immunofluorescence microscopy (**c**). Staining cortical actin (*green* in **c**) helps to define the cell borders. The protoplasmic (P) and extracellular (E) fracture faces have been labeled in the replica of the gap junction plaque (in **b**). Nucleus = n. Bars: 100 nm in (**a**), 60 nm in (**b**), and 10 μm in (**c**). (**a** from ref. [58] and **b** from ref. [206])

While the first transmission electron microscopic images of gap junction plaques were acquired in the early 1960s [29–31], it was not until 1972 that the first ultra-structural evidence for the endocytosis of the gap junction plaque and annular gap junction vesicle was reported [32]. Annular gap junction vesicles were described as double-membrane vesicles surrounding a central lumen seen in the cytoplasm of cells [25, 33] (Fig. 2a, marked with arrowhead). The 2-4 nm “gap” seen between the double membranes of the vesicle was the same width as that measured between the membranes of the gap junction plaque [34–36]. The gap junction plaque was named as a result of the presence of this gap [37–40], and thus the annular gap junction also was named based on that consistent narrow gap and its annular morphology. Annular gap junction vesicles have also been termed connexosomes [41]. The appearance of the annular gap junction vesicle membranes, as well as the gap junction plaque membranes, is determined by the fixation and staining methods used to prepare the samples. Specifically, a three-layered gap junction membrane was described after osmium tetroxide fixation followed by lead citrate staining [42]. A five-layered gap junction membrane was reported after potassium permanganate fixation followed by uranyl acetate and lead citrate staining [29, 30]. A seven-layered membrane was detected following fixation with glutaraldehyde-osmium tetroxide and stained en bloc with uranyl acetate before alcohol dehydration [39]. These early ultra-thin section structural analyses unmistakably characterized gap junctions as novel cellular structures and unambiguously differentiated gap junctions from another cell-cell junction type, tight junctions [43].

### Freeze fracture electron microscopy

The first freeze fracture electron microscopic report describing annular gap junction vesicles was published in 1973 [44]. With freeze fracture, the cell membrane is split in the hydrophobic plane at the level of contact between the acyl chains of the phospholipid molecules that comprise the two leaflets of the membrane bilayer [45]. This results in a protoplasmic (P)-fracture face (which represents the outer leaflet of the plasma membrane bilayer that is still adherent to the underlying cytoplasm as observed from the extracellular space looking inward) and an extracellular (E)-fracture face (which refers to the inner leaflet of the fractured membrane bilayer that was adjacent to the extracellular space as seen looking outward from the cytoplasmic space) (Fig. 2b). Since the fracture face can jump from within one membrane to within the other membrane (as is the case in the gap junction plaque shown in Fig. 2b), freeze fracture allowed unambiguous identification of gap junction channels because they traversed both plasma membranes and gap junction channel halves (connexons) were present on both replicas [46]. The annular gap junction vesicle P- and E-fracture face appearance was the same as that seen for the gap junction plaque [47–49]. Specifically, freeze fracture disclosed aggregates of 8.5 nm particles on the P-fracture face and clusters of pits on the E-fracture face of the cytoplasmic vesicles [47, 49]. The annular gap junction vesicle however was distinguished from the plaque by its obvious location within the cytoplasm and its vesicular appearance [49]. Based solely on the early TEM and freeze fracture images, it was hypothesized that gap junction plaques were engulfed into one of two contacting cells [32, 33, 48, 49], but the definitive proof was yet to come.

It should be noted however, that in early years, the existence of annular gap junction vesicles was met with controversy. Some investigators suggested that the profiles seen in TEM were only cross sections through invaginations from the cell surface [50, 51]. However, meticulous serial sectioning through cells provided ultra-structural proof that there was a lack of continuity of the annular gap junction vesicle profile with the cell surface and thus confirmed that at least some of the observed structures were truly isolated vesicles within the cytoplasm [32, 44, 52].

### Lanthanum infiltration

Further confirmation for the existence of annular gap junction vesicles rather than cross-sections of gap junction membrane invaginations came from lanthanum infiltration studies, which were used to demonstrate that the 2-4 nm “gap” of the annular gap junction membrane did not fill with lanthanum [52]. The lack of lanthanum in the “gap” between the inner and the outer membranes of the annular gap junction vesicles, thus confirmed that they were vesicles within the cytoplasm and not invaginations of the cell plasma membranes.

Annular gap junctions were found in a number of different cell types (ovarian granulosa cells, SW-13 adrenocortical tumor cells, epithelial cells, uterine cells, etc.) [33, 48, 49, 52–55] and investigators hypothesized that their formation was influenced by extracellular factors including toxins [41], viral infection [56] and hormonal treatments [25, 54]. The detection of annular gap junctions required highly skilled TEM and freeze fracture sample preparation and careful, laborious microscopic observations. The early studies of the distribution and changes in annular gap junction vesicles were therefore limited by the time and difficulty of obtaining the sample size needed for quantitation. New methodologies were needed that allowed for the rapid and accurate identification of annular gap junction vesicles if information on the tissue distribution and mechanisms of regulation were to be obtained. Such new methodology arrived with the isolation, characterization and production of antibodies against the gap junction channel connexin proteins [2, 57].

### Immunofluorescence microscopy (two and three-dimensional analyses)

With the availability of antibodies directed against the various connexin family members, it was possible to use immunofluorescence microscopy to demonstrate the vast tissue distribution of annular gap junction vesicles. Immunofluorescence, compared to TEM or freeze fracture, is a relatively straightforward and inexpensive method for detecting gap junction plaques and annular gap junction vesicles. By using an anti-connexin antibody and a secondary antibody (which has been conjugated to a fluorophore) it was possible to visualize annular gap junctions with a fluorescence microscope [24, 55, 58–61] (Fig. 2c, marked with arrowhead). Furthermore, colocalization procedures have been used to evaluate connexin-associated proteins and to predict the role of these proteins in annular gap junction processing [18, 58, 59, 61, 62]. Annular gap junction vesicles, detected with immunofluorescence, were generally > 0.5 μm in size [61] and were distinguished from either secretory vesicles [63], which generally are less than 200 nm in diameter, or from aggregated fluorescent material based on size and the annular staining morphology of the puncta [64, 65].

**Fig. 3:**
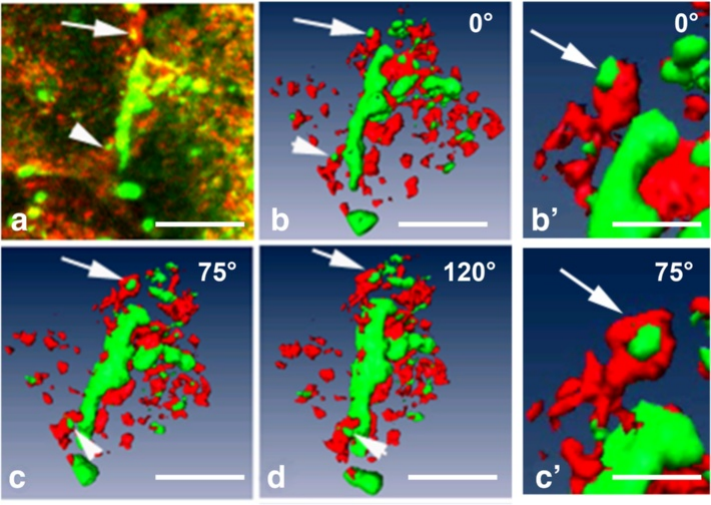
Immunocytochemistry (**a**) and 3D-volume reconstructions (**b**-**d**) demonstrating in detail the association of clathrin (*red*) with a Cx43 (*green*) gap junction plaque. An area of the gap junction plaque can be appreciated in the rotated views as a bud that likely has not yet detached from the plaque (**a**-**d** *arrowheads*). It can be confirmed in the rotated views that an annular gap junction is intimately associated with clathrin (*arrows* **a**-**d**). A rotation around the Y-axis in the 3D-reconstruction images allows more information to be obtained on the morphology of the gap junction structures as well as their relationship to clathrin. The images seen in (**b**) and (**c**) (*arrows*) have been enlarged (**b**'-**c**'). The 3D-reconstruction was rendered with the Amira 3D Software for Life Sciences, FEI™ (Hillsboro, Oregon). Bar: 5 μm in (**a**-**d**), 1.5 μm in (**b**’, **c**’)

One disadvantage of two-dimensional immunofluorescence microscopic analyses is that false-positive colocalizations can occur when two molecules are in visual alignment (superimposed in the image) but are not in contact [66]. Three-dimensional confocal analysis is a major refinement of the immunofluorescence microscopic technique and it allows assessment of the co-localization (molecular interaction) of selected molecules. That refinement consists of obtaining confocal “z-stacks”, which are multiple confocal images spaced equally in the “z” axis, in order to generate orthogonal x-z and y-z side views. This also allows for the generation of a 3D volume-view in which the positions and proximity of the molecules of interest can be ascertained (Fig. 3). The ability to view the cellular components from different angles provided by the rotation of the re-constructed volume view permits a highly critical analysis of "true" colocalization [58, 62, 67]. In Fig. 3, as an example, the association of clathrin with the gap junction plaque and annular gap junction vesicles has been displayed in several different angles. This allows for a detailed analysis of the morphology of the gap junction structures and their interactions with clathrin. A methods article, which describes in detail immunohistochemical procedures to analyze gap junction plaques and annular gap junction vesicles is forthcoming [68].

### Quantum dot immuno-electron microscopy

**Fig. 4:**
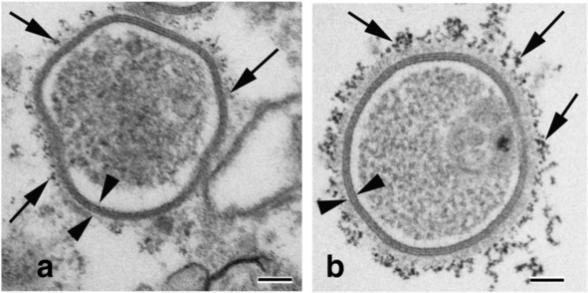
Quantum dot immuno-electron microscopy demonstrating annular gap junction vesicles decorated with phosphorylated Cx43 (**a**) and clathrin (**b**) (*arrows*). Note the characteristic annular gap junction double-membrane, which helps to distinguish the annular gap junction vesicle from other membraned cellular structures (see *arrowheads*). Bars: 100 nm

Annular gap junctions and their associated proteins, detected with immunofluorescence have been accurately identified and correlated with quantum dot immuno-electron microscopy techniques. With quantum dot immuno-electron microscopy techniques, the characteristic gap junction membrane ultrastructure can be easily discerned and used to positively identify annular gap junction vesicles and their associated proteins (Fig. 4). As seen in Fig. 4a, for example, phosphorylated Cx43 can be observed associated with the annular gap junction vesicle while in Fig. 4b, clathrin can be seen decorating an annular gap junction (marked with arrows in both images). The annular gap junction can be positively identified in these images by the presence of the characteristic pentalaminar membrane (marked with arrowheads). A methods article which describes in detail quantum dot probes and procedures for imaging gap junction structures is forthcoming [68].

### Live cell imaging

**Fig. 5:**
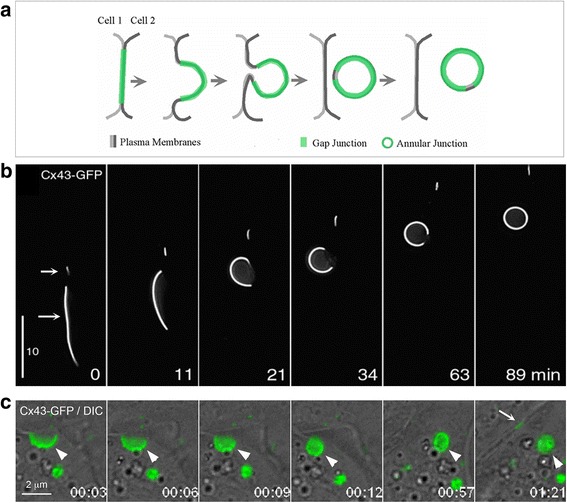
Internalization of a gap junction plaque. **a** Schematic depicting the internalization of a complete gap junction. The process leads to the formation of an annular gap junction (AGJ) vesicle in the cytoplasm of one of the coupled cells. **b**, **c** The process schemed in (**a**) imaged by time-lapse microscopy in Cx43-GFP expressing HeLa cells (only fluorescence shown in (**b**), merged fluorescence and DIC channels shown in (**c**)). Note that the small gap junction plaque in (**b**) (depicted by the upper arrow) does not internalize and remains in the plasma membrane while the large gap junction (depicted by the lower arrow) invaginates into the left cell of the coupled pair and forms an annular gap junction; and that in (**c**) a new gap junction (depicted with arrow) forms at the location were the previous internalized gap junction plaque (depicted with arrowhead) was localized. Time in c is in hours:minutes. (B.N.G. Giepmans and C. Lehmann recorded time-lapse movie sequences shown in (**b**) and (**c**), respectively when working in the Falk lab.)

**Fig. 6:**
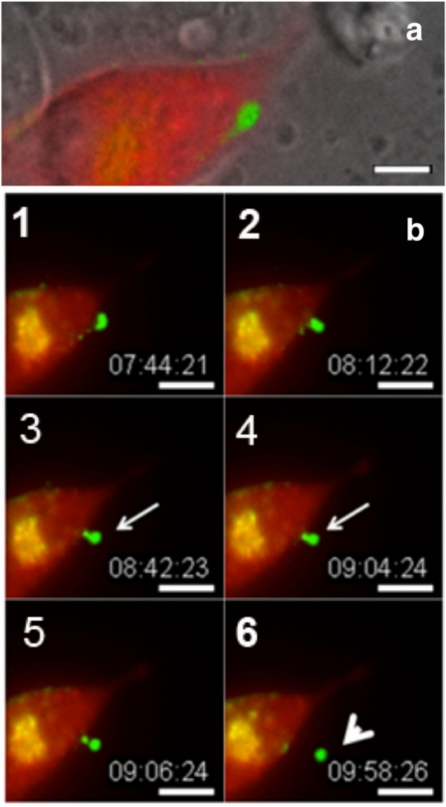
Time-lapse image sequence of cells expressing Cx43-GFP. As evident from the phase/fluorescence overlay image (**a**) one of the two contacting cells is expressing RFP-tagged clathrin (*red*). In the corresponding montage of these cells, the gap junction plaque invaginates to form a gap junction bud (*arrow*), which is then scissored from the membrane to produce an annular gap junction vesicle (*arrowhead*) (**b**, 1-6). Time is in hours:minutes:seconds. Bar: 15 μm. (From ref. [58])

The field advanced further by the isolation of the jellyfish green fluorescent protein (GFP) [69], the coupling of GFP to connexins, and the expression of connexin-GFP fusion proteins in cells [63, 65, 70, 71]. The connexin-GFP fusion protein has been used for live cell imaging studies to investigate connexin protein dynamics [63, 65, 71]. Investigators applying live cell imaging [63, 65, 71, 72] have confirmed the suggestions of the earlier morphologists that annular gap junction vesicles indeed result from the internalization of gap junction plaques into one of the two contacting cells. Important to the discussion of annular gap junction vesicles is that clusters of gap junction channels can be removed from relatively small areas of the plaque, and that an entire gap junction plaque can be internalized to form annular gap junctions [58, 61, 73] (Figs. 5 and 6). The size of the formed annular gap junction was seen therefore to vary depending on the size of the gap junction plaque or the portion of the plaque that was internalized. The observations made with live cell imaging have contributed to the understanding of gap junction dynamics and the characterization of the numerous proteins that were demonstrated to be involved in their formation, fission into smaller vesicles and their degradation. A methods article describing in detail live-cell probes and procedures to image gap junctions and annular gap junctions in living cells is forthcoming [74].

## Annular gap junction formation

### Dynamic changes occurring in gap junction plaques during annular gap junction formation

**Fig. 7:**
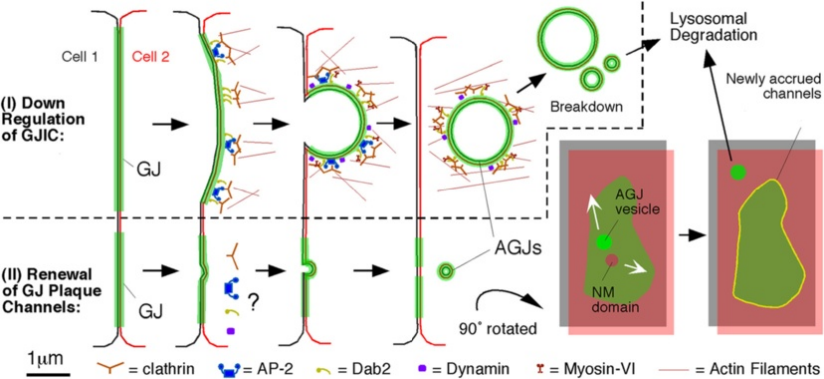
Schematic representation of the pathways that lead to the internalization of entire gap junction plaques (I) and of central plaque portions (II), annular gap junction formation, fission, and degradation. Whether clathrin and clathrin-accessory proteins are involved in the internalization of small gap junction vesicles shown in (II) has not been determined, however is likely based on our EM analyses. Accrual of new channels (yellow line circumscribing the green gap junction plaque) is accompanied by the simultaneous internalization of central plaque portions consistent with previously published observations [63, 72]. Clathrin and accessory proteins are shown in patches in accordance to the appearance of clathrin on gap junction plaques [61], and the current thinking that clathrin may provide a scaffold for directed actin assembly, facilitating internalization of large structures such as gap junctions, viruses, and pathogenic bacteria [207, 208]. The manner in which clathrin and accessory proteins are drawn still remains somewhat speculative. NM = Connexin-free junctional membrane domain. (From ref. [73])

**Fig. 8:**
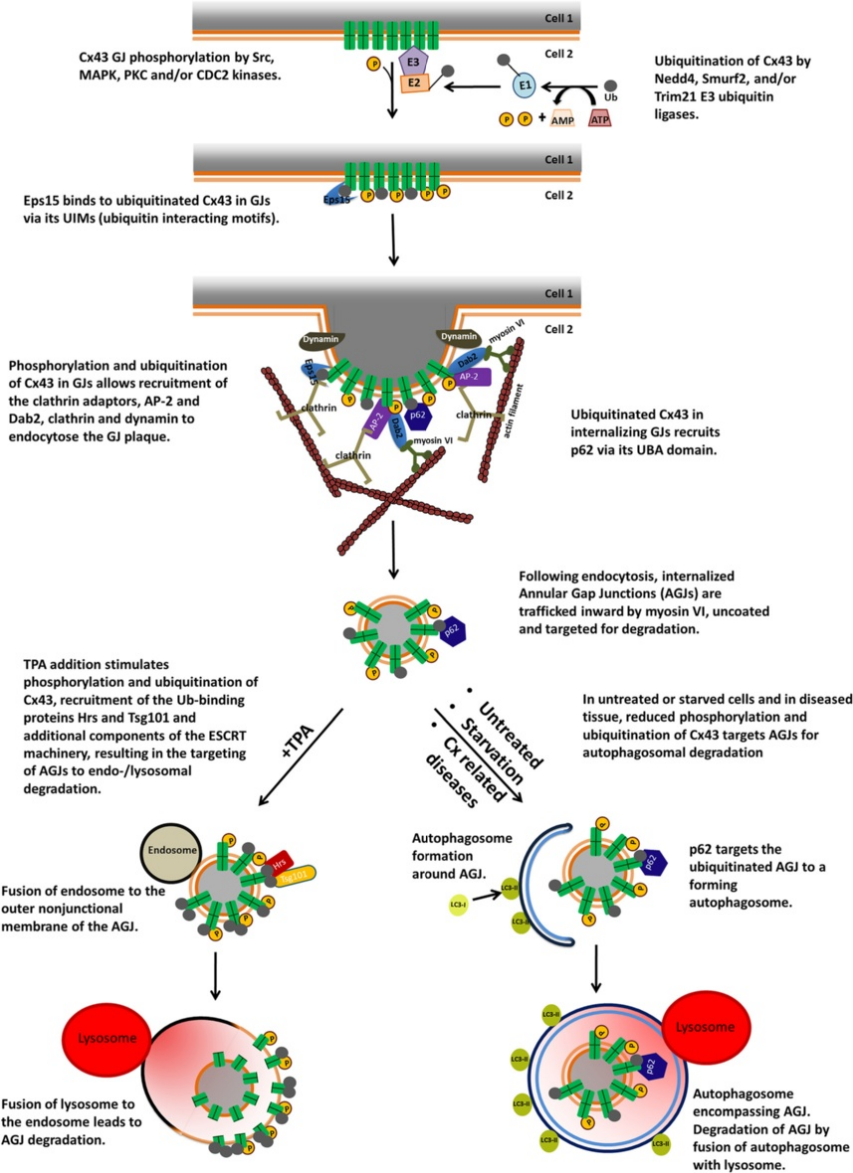
Schematic representation of the signals and players that participate in the steps that lead to gap junction internalization, formation of annular gap junctions in the cytoplasm of the acceptor cell, and annular gap junction degradation through the phago-lysosomal (*bottom right*) or the endo-lysosomal (*bottom left*) pathway based on published studies. Abbreviations are: AGJ, annular gap junction; CLASPs, clathrin-associated sorting proteins; ESCRT, endosomal sorting complexes required for transport; GJ, gap junction; p62, sequestosome 1/SQSTM1; UBA, ubiquitin-associated domain; UIMs, ubiquitin-interacting motifs. (From ref. [182])

The internalization of the gap junction plaque membrane is a critical step in the removal of gap junction plaques. Based on time lapse image analyses the gap junction plaque internalization process first involves the formation of a shallow curvature of the gap junction plaque [58, 61]. This invaginated area then deepens to form a U-shaped projection of gap junction plaque membrane. Over time, the U-shaped invagination develops into a full bud-like membrane area, which remains attached to the plasma membrane by a thin neck. The bud is subsequently detached from the plasma membrane to form a cytoplasmic annular gap junction vesicle [58, 61] (Figs. 5, 6 and 7). The frequency of gap junction plaque internalization and subsequent annular gap junction vesicle degradation is thought to impact a number of pathological conditions, including cancer and ischemia [48, 75, 76], and further may also be critical to numerous cellular functions, such as cell migration, proliferation, and wound healing [24, 77]. The steps in the gap junction plaque internalization process that result in the formation of annular gap junction vesicles is a highly regulated phenomena which likely requires coordinated participation of numerous signaling machineries (Figs. 7 and 8).

### Molecular signals that initiate annular gap junction formation

**Fig. 9:**
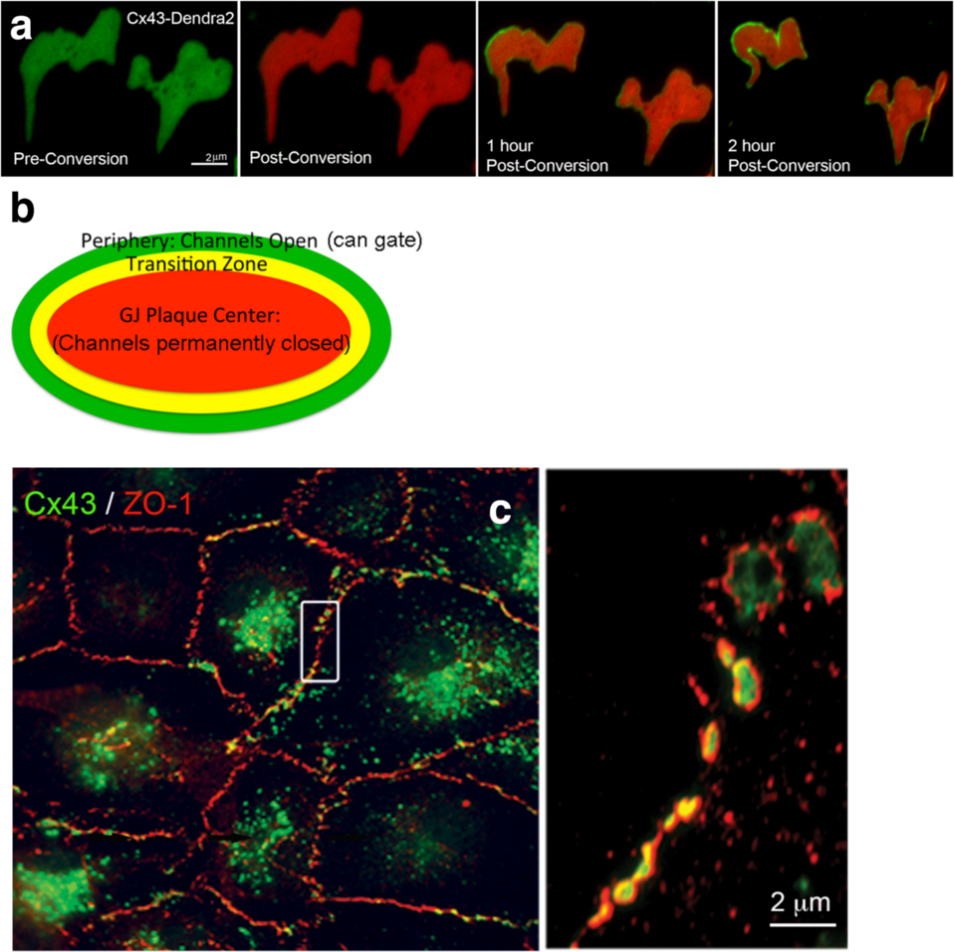
Gap junction plaque assembly and structure. **a** Photoconversion of Cx43-Dendra2 reveals accrual of newly synthesized gap junction channels (non-converted, *green*) along the outer edge of gap junction plaques (permanently photoconverted from green to red; shown for two gap junction plaques viewed en face/onto the plaque surface). (From ref. [73].) **b** Schematic model of a gap junction depicting our hypothesized plaque organization. **c** Cx43 gap junctions (*green*) colocalizing with the scaffolding protein, ZO-1 (*red*), generating a typical staining along the rim of gap junction plaques [130], shown in fixed endogenously Cx43 expressing primary pulmonary artery endothelial cells (PAECs). The boxed area is shown enlarged on the right. (From ref. [131])

While the structure and formation of annular gap junctions is now well documented [33, 55, 59, 61, 78], still very little is known about the molecular signals that initiate gap junction internalization. Previous studies have shown that gap junction channels of a gap junction plaque appear to turn over continuously, an observation that correlates well with the short half-life of only 1–5 h observed for connexins and gap junctions in situ and in cultured cells [63, 72, 73, 79–81]. Numerous studies have shown that newly synthesized channels accrue along the outer periphery of gap junction plaques while older, and likely non-functional channels are simultaneously internalized from central plaque regions [58, 63, 72, 73, 82, 83] (Fig. 9a). Alternative methods of gap junction plaque assembly also have been suggested in which connexons accrue and dock throughout plaques. In addition, the fusion of dispersed or clustered connexons and gap junction channels by lateral movement may exist as well [84–87]. Interestingly, a number of recent reports suggest that only a small portion of gap junction channels in a gap junction plaque is open and functional, while the majority seem to be permanently closed [15, 88–90]. These observations would correlate with a model that predicts that only newly accrued channels (in the plaque periphery) are functional, while older more central plaque portions are non-functional, remaining closed until they are removed via internalization (Fig. 9b). However, in addition to internalizing non-functional, central plaque portions [65, 73], cells have been observed to internalize entire gap junction plaques while other gap junctions in these cells remained stable in the plasma membranes [59, 61, 67, 91, 92] (Fig. 5b). As described in detail above, several studies have shown that gap junction internalization utilizes components of the endocytic clathrin machinery [58, 59, 61, 82, 93, 94] (Figs. 7 and 8). As shown for a number of proteins, including gap junction connexins, clathrin does not interact with its cargo directly but indirectly via adaptors. Three different clathrin associated sorting proteins (CLASPs) have been identified to recruit clathrin to Cx43, the classical plasma membrane clathrin adaptor protein complex, AP-2, the alternative clathrin adaptor protein, Dab2 (disabled 2) [61], and the ubiquitin-interacting CLASP, Eps15 (Epidermal growth factor receptor substrate 15) [95]. Depleting cells of these adaptors by RNAi significantly reduced gap junction internalization [93, 95], which indicates that these adaptors are able to recruit clathrin to Cx43 for gap junction internalization. Three adaptor-protein binding sites (canonical tyrosine- based sorting signals of the type ‘YXXΦ’, where Φ is a bulky hydrophobic amino acid) termed S1, S2 and S3 (Y^230^VFF^233^, Y^265^AYF^268^, Y^286^KLV^289^) were identified in the connexin C-terminus (Cx43-CT), two of which (S2 and S3) were found to function cooperatively as AP-2 binding sites [96]. The S3 binding site is part of a previously identified internalization-relevant region termed proline-rich region [97]. Mutating or deleting the adaptor-protein binding sites abolished Cx43/adaptor protein/clathrin interaction, which results in increased gap junction size, longer Cx43 protein half-lives, and most importantly abolished gap junction internalization [95, 96]. From these observations, it seems clear that internalizing gap junction plaques and non-functional gap junction plaque portions (presumably central regions) need to interact with components of the endocytic machinery (CLASPs/clathrin), while stable, functional, gap junction plaques and gap junction plaque portions (presumably peripheral regions) do not. Such a selective Cx/CLASP/clathrin interaction would require significant structural differences in the connexins of internalizing gap junction plaques and plaque portions compared to their plasma membrane dwelling counterparts. What are these structural differences in internalizing versus stable gap junction-localized connexins that mediate CLASP/clathrin access and binding in such a precise and sophisticated manner? A number of connexin modifications including (1) connexin phosphorylation, (2) ubiquitination and (3) ZO-1-binding are known to directly or indirectly influence gap junction mediated intercellular communication (GJIC) and thus may also influence gap junction internalization and annular gap junction vesicle formation.

### Connexin phosphorylation

Cx43 and many other connexins are phospho-proteins and most, if not all steps in their ‘life cycle’ are regulated via phosphorylation/de-phosphorylation by various kinases [98–101]. At least 11 serine and two tyrosine residues located in the Cx43-C-terminus (CT) are known to be phosphorylated in vivo which result in either GJIC up-regulation (S325, S328, S330, S364/365, S373) or down-regulation (Y247, S255, S262, Y265, S279/282, S368) [98, 100, 101]. Serine phosphorylations that decrease GJIC are mediated by PKC (S368) [100, 102, 103], mitogen activated protein kinase (MAPK) (S255, S262, and S279/S282) [104–106], and by the cell-cycle dependent kinase CDC2 [91, 107–109], while tyrosine residues are phosphorylated by Src [110, 111]. Recently, MAPK- and PKC-mediated phosphorylation of Cx43 on serines 252, 262, 279/282, and 368 was directly linked to clathrin recruitment and gap junction endocytosis in mouse embryonic stem cells and in primary pulmonary artery endothelial cells (PAECs) in which gap junction endocytosis was induced by treatment with the growth factors, EGF and VEGF, respectively [112, 113]. Phosphorylations by both MAPK and PKC kinases were found to be essential for gap junction internalization [112, 113]. In addition, two other studies report that mutation of amino acids S279 and S282 into phospho-dead alanine residues increased Cx43 protein stability upon treatment with EGF [114] and that activation of PKC resulted in the phosphorylation of Cx43 S368 which caused gap junction internalization and degradation [88]. Moreover, the Mehta lab published that phosphorylation on S279/282 regulates Cx43 gap junction endocytosis in pancreatic cancer cells [115]. Together, these studies are consistent with the suggestion that phosphorylation/de-phosphorylation events on at least two different sites in the Cx43-CT (S368 and S279/282) by two different kinases (PKC and MAPK) are directly linked to clathrin recruitment and gap junction internalization.

De-phosphorylation on Ser365 (initially phosphorylated by PKA) has been shown by the Lampe and Sorgen labs to be essential for allowing phosphorylation of Ser368 (termed ‘gate-keeper’ event) [116]. Interestingly, Ser365 de-phosphorylation causes a major conformational alteration of the Cx43 C-terminus that involves the upstream region around amino acid 280 [116, 117], the region we now know harbors the S2/S3 AP-2/clathrin binding sites. Thus, de-phosphorylation of Ser365, required for subsequent phosphorylation of Ser 368 and its link to the conformational alteration of the Cx43-CT, is likely to also play a direct role in gap junction internalization.

### Connexin ubiquitination

Another post-translational modification known to occur on Cx43 is ubiquitination. Ubiquitin (Ub) is a small, 8.5-kDa protein that is covalently attached to a lysine on a target protein by an enzyme cascade consisting of E1 (Ub-activating), E2 (Ub-conjugating) and E3 (Ub-ligase) enzymes [118]. A single Ub (mono), multiple mono, or a chain of Ubs linked together (poly Ub) can be attached to a target protein. All 7 lysines in the Ub polypeptide (K6, 11, 27, 29, 33, 48, 63) are capable of forming structurally precise linkages to subsequent Ubs [119], and all serve different and specific cellular functions. Ubiquitination of Cx43 has been described for 20 years, and its role in signaling proteasomal Cx43 degradation has been well established [120]. However, Ub’s role in the internalization and degradation of gap junctions has only recently been studied [16, 22, 23, 95, 121]. We still know very little about the types of ubiquitination that occur in gap junction plaques, how many Ub moieties are linked to Cx43 polypeptides in gap junctions, how many connexin polypeptides in a gap junction channel need to be ubiquitinated (just one, or all 6 on one side of the plaque), and to which lysine residue(s) the Ub(s) is/are linked. So far, the few published results indicate that multiple mono-Ubs are attached to connexins [22, 95], and very recently that K63-poly-ubiquitination also associates with connexins [122]. K63-linked poly-ubiquitination is known to signal intracellular trafficking, endo-lysosomal, and phago-/lysosomal degradation [123, 124]. This would suggest that Cx43 K63-poly-ubiquitinatination might also play a role in gap junction internalization. Lysine 303 in the Cx43-CT was identified in a proteome-wide survey of potential Ub sites [125] making this residue the most likely candidate for K63 poly-ubiquitination.

### ZO-1 binding and release

A large number of structural and cytoplasmic regulatory proteins are now known to interact with Cx43, predominantly at its C-terminus (reviewed in [98, 126]). One of these regulatory proteins is a MAGUK (membrane-associated guanylate kinase)-family member known as ZO-1 (zonula occludens-1). It is a well-characterized ubiquitous plasma membrane-associated scaffolding protein that has been shown to be involved in regulating gap junction plaque assembly. ZO-1 directly interacts with Cx43 and other connexins through its PDZ-2 domain [98]. The last 4 residues of Cx43 insert into a binding pocket on the PDZ-2 domain [127, 128]. Two PDZ-binding consensus sequence motifs, class 1 (-X-**S/T**-X-**V/I/L**-COOH) and class 2 (-X-**V/I/L**-X-**V/I/L**-COOH) have been identified that mediate PDZ-domain/binding protein interactions [129]. The minus one and the minus three position residues (bold) are of particular importance. The last four amino acid residues of the majority of the connexins contain these consensus sequence motifs suggesting the interaction of these connexins with ZO-1 [98]. Interestingly, ZO-1 only localizes to the periphery of Cx43 gap junction plaques, generating a typical rim staining [130, 131] (Fig. 9c). More recently, Rhett and colleagues showed that Cx43 connexons are bound to ZO-1 when they dock with connexons from apposed cells in the vicinity of plaques (a region termed the perinexus) and that ZO-1-binding down-regulates the rate of channels that are added to GJ plaques [83]. Dunn and Lampe recently showed that Cx43/ZO-1 binding is regulated via phosphorylation/de-phosphorylation of S373 by Akt kinase [132]. This finding correlates with the earlier observation of Chen and colleagues which implemented peptides corresponding to the Cx43-CT with serines 372 and 373 mutated into the phosphomimetic residue glutamic acid (E) to investigate Cx43/ZO-1 interaction in in vitro protein/protein binding assays [133]. Cx43-S373 that is phosphorylated by Akt kinase early on in the secretory pathway [134, 135] was found to be de-phosphorylated in Cx43-subunits that bind ZO-1 [132]. These results suggest that ZO-1 interaction with connexins in gap junctions, in addition to phosphorylation and ubiquitination, plays a central role in gap junction plaque turnover.

**Fig. 10:**
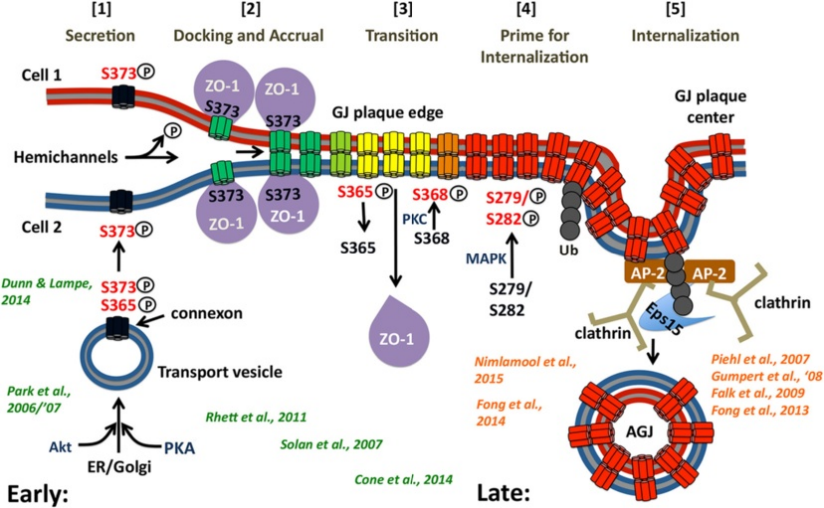
Scheme depicting the molecular signals in the Cx43-C-terminal domain hypothesized to regulate gap junction assembly and internalization based on our own (*orange*) and colleagues’ (*green*) findings. Steps [1 – 5] trigger and coordinate the transition from functional (*green*) into non-functional, internalization-prone gap junction channels (*yellow*, *orange*) that then are primed via post-translational modifications to allow interaction with clathrin components to mediate their internalization (*red*). The color scheme of the channels corresponds to the schematic gap junction shown in Fig. 9b

Based on, and backed significantly by the observations made by us and others described above, we pieced together a working model depicting the molecular alterations that regulate GJ assembly and turnover: The model predicts that a series of consecutive phosphorylation/de-phosphorylation events on well-known C-terminal Cx43 amino acid residues including Ser373, Ser365, Ser368, and Ser279/S282 known to be phosphorylated by Akt, PKA, PKC, and MAP kinases, respectively, regulate [1] forward trafficking of connexons to the plasma membrane (secretion), [2] connexon docking and channel accrual, and [3] transition of functional into permanently closed gap junction channels that then are [4] ‘primed’ to interact with clathrin to [5] mediate their endocytosis. These 5 steps also involve and require Cx43-ubiquitination and ZO-1 binding and release. All steps, including the critical posttranslational modifications are shown schematically in Fig. 10. The 5 steps trigger and coordinate the transition from functional (green) to internalization-prone GJ channels (yellow, orange) that then can interact with clathrin components to internalize GJ channels (red). The 5 steps are as follows: [1] Early during secretion (ER, Golgi), newly synthesized connexons are phosphorylated by Akt kinase on S373 (and potentially S372) to prevent premature ZO-1 binding. These connexons are trafficked to the plasma membrane and may function as hemi-channels [13, 14] as S373 phosphorylation prevents their docking into double-membrane spanning gap junction channels. [2] Upon de-phosphorylation of S373 in the vicinity of gap junction plaques (the perinexus) they bind ZO-1, dock, and accrue to the periphery of gap junction plaques. [3] When gap junction channels age (permanently close) and move inward towards plaque centers, de-phosphorylation of S365 is initiated to allow phosphorylation of S368 to transition ZO-1-bound into ZO-1-unbound gap junction channels. [4] Conformational changes of the Cx43-CT triggered by de-phosphorylation of S365 allow MAPK to access and phosphorylate S279/282 (and potentially also S262 and S255), and E3-ubiquitin ligases (Nedd4, Smurf2, Trim21, Wwp1) [95, 136–138] to bind to and ubiquitinate Cx43 (potentially on K303). [5] These protein modifications then enable CLASPs (AP-2, Eps15) to access and bind to Cx43 to recruit clathrin and invaginate the central gap junction plaque portion. Dynamin (directly or indirectly) then likely scissors the invaginated gap junction bud from the plasma membrane [58, 82, 93, 94]. Acute internalization of entire gap junction plaques (e.g. induced by thrombin, endothelin, EGF, VEGF, TPA-treatment [88, 112, 113, 131, 139], or following hormonal changes [140]) may be achieved in a similar way by initiating ZO-1 displacement on the endocytic side of gap junction plaques as observed by Gilleron and colleagues [67] and Baker and colleagues [131].

**Fig. 11:**
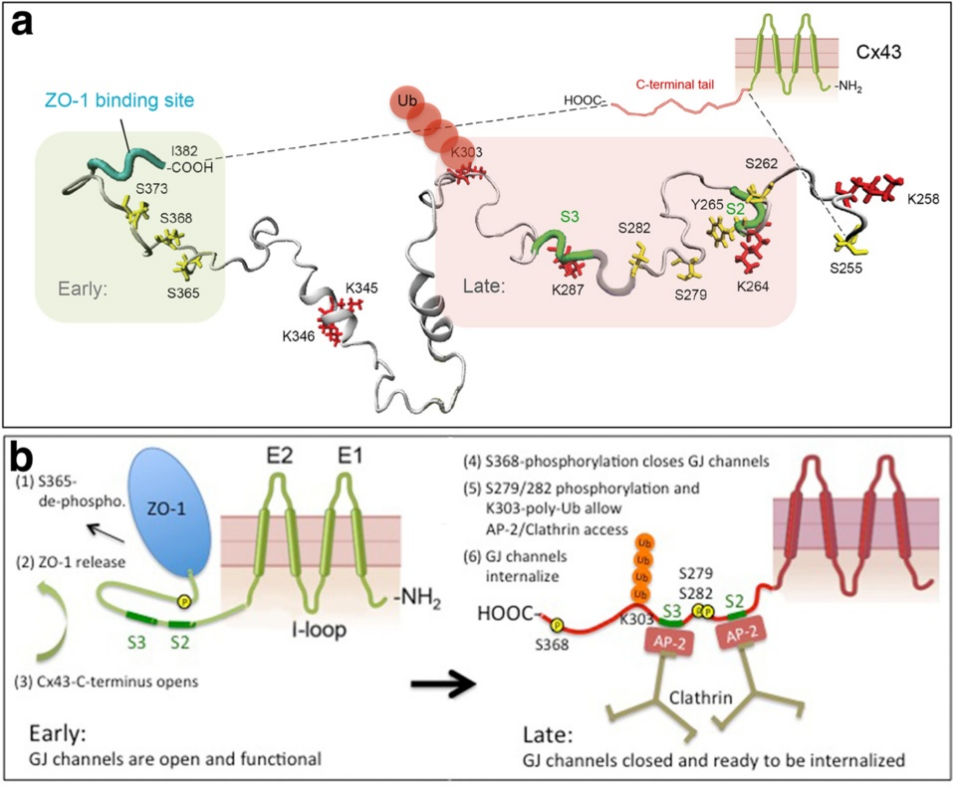
Scheme depicting how access of clathrin to Cx43 might be regulated. **a** Interestingly, all proposed Cx43 modifications relevant to Cx43 gap junction internalization cluster into two domains, ‘early’ occurring on residues located juxtaposed to the C-terminal ZO-1 binding site (*shaded green*), and ‘late’ occurring on residues located juxtaposed to the S2, S3 AP-2 (Eps 15)/clathrin binding sites (shaded red). The lowest energy 3D solution NMR structure of the Cx43-CT revealing the location of critical residues solved by Sorgen and colleagues [117] is shown. **b** We propose that a conformational change of the Cx43-C-terminal domain (CT) triggered by serine 365 de-phosphorylation [116] opens up the Cx43-CT allowing MAPK to access and phosphorylate S279/282 (and eventually also S262 and S255); and E3-ubiquitin ligases to bind to and ubiquitinate Cx43 (presumably on lysine 303) to promote AP-2 (and/or Eps15) to access the YXXΦ -binding motifs (S2, ^265^YAYF^268^; S3, ^286^YKLV^289^), recruit clathrin and internalize gap junctions/central gap junction plaque portions

Note that the 5 transition steps described above group into two phases, “Early” (green) and “Late” (red) that are spatially separated on the Cx43 C-terminus (Fig. 11a). Early events (including S373 phosphorylation/de-phosphorylation, ZO-1 binding/release, S365 de-phosphorylation and S368 phosphorylation) occur on the far C-terminal portion of the Cx43 C-terminus juxtaposed to the ZO-1 binding site, while later events (S279/S282 phosphorylation, ubiquitination) occur further upstream around the CLASP/clathrin binding sites. Critical to our hypothesis is the above described observation published by the Lampe and Sorgen labs [116] that Ser365 de-phosphorylation (a prerequisite for S368 phosphorylation) causes a major conformational alteration of the Cx43 C-terminus that involves the upstream region that harbors the S2/S3 CLASP/clathrin binding sites. Based on this observation we hypothesize that the Cx43 amino acid region around the clathin binding site is not accessible to CLASPs (based on folding and steric hindrance) in connexins of functional gap junction channels, while the region is rendered accessible (unfolded) to CLASPs and clathrin via phosphorylation and ubiquitination in gap junction channels that are to be endocytosed (Fig. 11b). MAPK-mediated phosphorylation (on S279/S282 and potentially on S262 and S255) and ubiquitination (presumably on K303) in the vicinity of the CLASP/clathrin binding sites may increase hydration and thus may further enhance CLASP/clathrin access. Alternatively, Cx43-ubiquitination could regulate binding of CLASPs, such as AP-2 versus Eps15. Eps15 is a clathrin-binding protein that interacts with the target protein via direct interaction of its ubiquitin-interacting motif (UIM) with the Ub-modification that is surface localized and accessible in K63-poly-Ub chains [141], while AP-2’s μ2-subunit interacts with tyrosine- and dileucine based amino acid sequence motifs [142] that are present within the Cx43-CT [96]. Such a shift from using tyrosine-/dileucine-based (AP-2) to Ub-based (Eps15) CLASPs has, for example, been described for EGF-receptor internalization and is believed to accommodate a switch from constitutive to acute receptor internalization [143]. [Note added in proof: During the typeset stage of this manuscript a study demonstrating that phosphorylation of connexin43 serine residues 279/282 increases affinity of the E3-ubiquitin ligase, Nedd4 by two-fold and thus appears to directly regulate Cx43-K63-polyubiquitination was published [144].]

Chen and colleagues Cx43-peptide/PDZ-domain binding assays also revealed that the Cx43/PDZ-2 interaction requires that two ZO-1 proteins undergo a structural dimerization known as “domain swap” as they bind two separate Cx43 C-terminal peptides. Such dimerization results in a structural assembly that is believed to make the PDZ-2/Cx43 interaction Cx43 specific [133]. Notably, these results indicate that at least two ZO-1 proteins will simultaneously interact with a connexon of a channel in a gap junction plaque. As ZO-1 is a very large, 220 kDa protein (almost the size of an entire Cx43-based connexon) dimerization forms a 440 kDa ZO-1 protein complex that dwarfs the size of the underlying bound connexon. Likely, this huge protein complex ‘shields’ the cytoplasmic surface of gap junction channels and thus will need to be removed from gap junction plaques before other proteins (kinases, phosphatases, E3-ubiquitin ligases) could gain access to gap junction channels and allow subsequent regulatory connexin modifications, including transforming them for gap junction internalization. Several other functions of ZO-1 associations with Cx43 have been described as well that range from channel formation to docking and lateral movement of the channels in the plasma membrane [83, 131, 145–147]. That ZO-1 most likely has functions in addition to regulating channel accrual is not surprising, as ZO-1 is a scaffolding protein and thus is likely to interact with many different proteins at various times.

As gap junction plaque assembly and internalization consists of several steps, it furthermore is not surprising that multiple players and post-translational modifications are involved to achieve these events. In addition, implementing a succession of different types of modifications likely increases specificity and flexibility, including aborting and reverting the chain of events in case such changed cellular conditions call for increased GJIC. Continued research will be essential to further substantiate this elegant and elaborate regulatory machinery described by our model. [Note added in proof: During the typeset stage of this manuscript a review article suggesting a similar model of the internalization of gap junctions was published lending further support to our gap junction internalization model presented here [148].]

### Annular gap junction translocation: actin and myosin-VI

In time-lapse recordings of living cells, internalized annular gap junction vesicles have been observed as translocating away from the plasma membrane and deeper into the cytoplasm [18, 61]. Staining Cx43-GFP transfected HeLa cells with anti-myosin-VI antibodies revealed a robust colocalization of myosin-VI with gap junction plaques that apparently were in the process of internalization (curved plaques), and with newly generated annular gap junction vesicles [61]. Myosin-VI did not colocalize with “stable” gap junction plaques (straight plaques apparently not in the process of internalization) or with Cx43-GFP containing secretory vesicles. In addition, actin filaments (stained with rhodamine-phalloidin) were observed to colocalize with Cx43-GFP gap junction plaques and annular gap junction vesicles [61]. This actin filament/Cx43-GFP annular gap junction colocalization was confirmed by ultra-structural analyses and is consistent with the well-documented role of actin in gap junction stabilization and internalization [33, 149–151]. That myosin-VI drives the translocation of annular gap junction vesicles from the plasma membrane into the cytoplasm was indicated by the effect of stabilizing or disrupting actin filaments with the known actin drugs, jasplakinolide and latrunculin A, respectively; and by overexpressing myosin-VI in Cx43-GFP expressing HeLa cells [61].

Myosin-VI is the only motor protein known to migrate toward the pointed (minus) ends (located peri-nuclearly) of actin filaments [152]. Myosin-VI has been described to function in translocating endocytic vesicles generated by clathrin-dependent endocytosis from the plasma membrane through the peripheral actin meshwork into the cell body [153, 154]. Myosin-VI can interact directly through its C-terminal globular tail with the C-terminal serine- and proline-rich region of the clathrin-adaptor, Dab2, and thus can link cargo and endocytic vesicles to actin filaments [153–157]. Since newly generated annular gap junction vesicles in general are much larger in diameter than normal endocytic vesicles (often > 0.5 μm compared to < 0.2 μm) it is not surprising that myosin-VI is recruited and aids in translocating annular gap junctions from the cell periphery deeper into the cell body. The involvement of the conventional plus-end directed actin-based motor, myosin-II, suggested to colocalize with internalized annular gap junctions [78] could not be confirmed in a later study by Piehl and colleagues [61] and its role, if any, in annular gap junction formation/processing remains unclear.

## Annular gap junction scission

### Fragmentation and budding

**Fig. 12:**
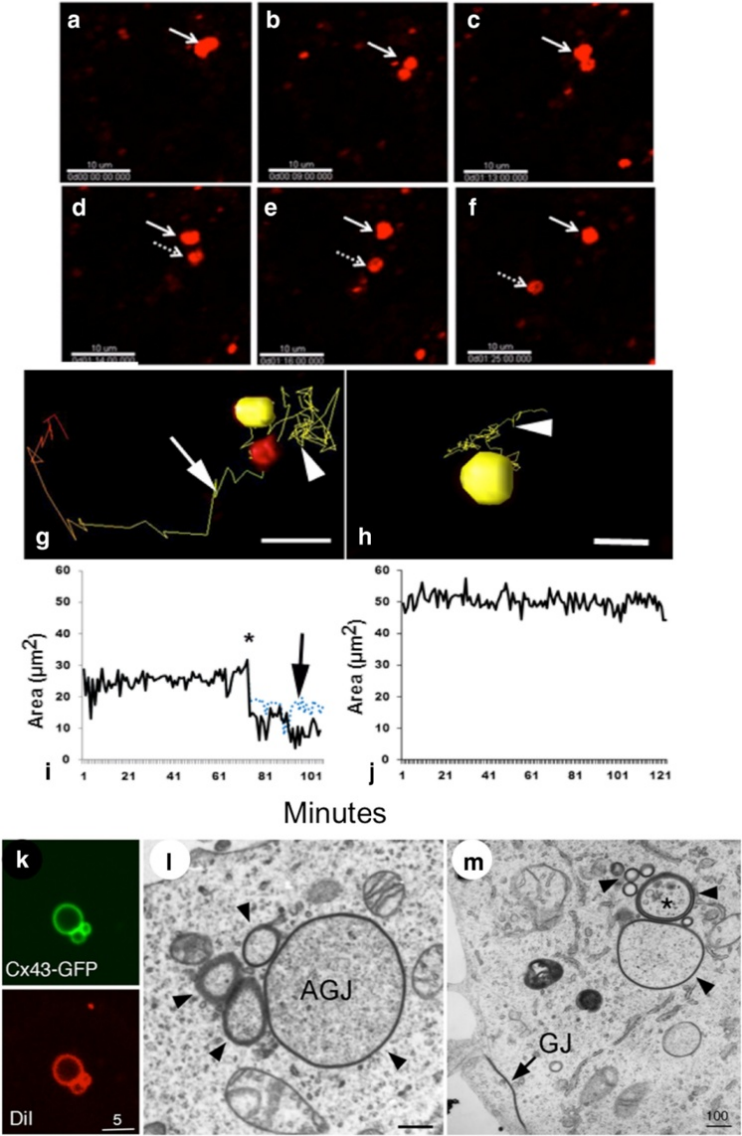
Annular gap junction vesicle fission. In the time-lapse imaging montage the splitting of an annular gap junction vesicle is demonstrated (**a**-**f**). Once the split occurs, the two annular gap junction vesicles (*arrow and dashed arrow*) move away from one another (**d**-**f**). The path and size of annular gap junction vesicles is depicted in the animated 3D reconstruction time-lapse tracking (**g**, **h**) and in the corresponding graphs (**i**, **j**). Note the yellow line depicting the annular gap junction movement path before (*arrowhead*) and after the split (*arrow*) (**g**) and the corresponding changes in size after fission (**i**). Inhibiting dynamin function blocks annular gap junction splitting (**h**, **j**). Clusters of annular gap junction vesicles, which would be consistent with the splitting/budding process, can be seen with fluorescence light microscopy in (**k**), and transmission electron microscopy (AGJ, marked with arrowheads in **l**, **m**) in the cytoplasm. Note the gap junction plaque (GJ) in the plasma membranes in (**m**). Membranes have been labeled with DiI (*red* in **k**). Bars: 10 μm (**a**-**f**), 5 μm (**g**, **h**, **k**), 100 nm (**l**, **m**). (**a**-**j** from ref. [58], and **k**-**m** from ref. [61])

Following internalization, annular gap junction vesicles have been reported to fragment or divide into smaller portions, or to bud smaller annular gap junction vesicles from a larger annular gap junction [58, 61] (Fig. 12). This fragmentation (scissoring) process results in the formation of typically appearing clusters of annular gap junctions visible in the cytoplasm in both fluorescence as well as EM images [58, 61, 158] (Fig. 12a-f and k-m). Indeed, by obtaining stacks of images, generating three-dimensional volume views, and collecting time-lapse microscopic images, annular gap junction scissoring has been confirmed [58, 61, 67]. In some cases near equal division of the annular gap junction was apparent (Fig. 12a-f). Both budding and annular gap junction scissoring resulted in a reduction of annular gap junction size. In the case of the small buds that are released from the annular gap junction vesicle, it is possible that some of these could be single rather than the typical double membrane that has been described for the annular gap junction vesicles [58]. Why annular gap junctions bud or scissor after their generation is currently not known, however it may prepare larger annular gap junctions for efficient degradation and potentially other uses. Dynamin is thought to facilitate the scissoring process.

### Dynamin’s role in annular gap junction formation and scission

Dynamin has been well documented to serve in the scissoring of numerous other types of non-junctional vesicles from membranes [159]. Specifically, dynamin has been shown to form a spiral around the neck of invaginated membrane during clathrin coated-pit formation [160–162]. Dynamin then tightens to constrict and eventually scissor the invaginated membrane from the surface [161, 163–167]. In the case of gap junction membrane scissoring, dynamin has been demonstrated to play a pivotal role in both the scissoring of the gap junction bud from the cell surface to result in annular gap junction vesicle formation [58, 61, 82, 93, 94] as well as in fission of annular gap junction vesicles to from smaller vesicles [58].

The evidence that dynamin facilitates scissoring of the gap junction plaque bud from the cell surface and thus annular gap junction formation is based on colocalization immunofluorescence studies in which dynamin was demonstrated to associate with Cx43-GFP gap junction plaques [58, 61], and on studies in which dynamin activity inhibition or reduction (by knocking down dynamin expression with RNAi, expression of a dominant negative dynamin mutant (K44A), or treatment with dynamin-inhibitory drugs such as Dynasore or GTP-γS in cells expressing Cx43-GFP) result in significantly fewer annular gap junction profiles [58, 61, 82, 93]. Furthermore, in the presence of dynasore, or in RNAi dynamin knockdown cells, the majority of gap junction buds failed to be released from the plasma membrane, in contrast to the relatively rapid release of these structures from plaques in the control populations [58].

By monitoring annular gap junction vesicles in control and dynasore treated cell populations and visualizing fission events by tracking the movement, morphological changes, and the size of individual annular gap junction vesicles over time, evidence has been provided that similar to its scissoring function in the release of gap junction plaque membrane buds, dynamin facilitates annular gap junction vesicle fission [58] (Fig. 12g-j). With time-lapse techniques, the average number of annular gap junction vesicles per hour seen to undergo fission was dramatically reduced in dynasore treated cell cultures compared to the number quantitated in control cell populations [58]. Consistent with these observations, immunocytochemical studies demonstrated dynamin at or near their equator area of some annular gap junctions [58, 82]. Based on dynamin’s site of location with annular gap junction vesicles, and the decreased number of fission events in the treated cell populations, it is suggested that dynamin plays a major role in the annular gap junction vesicle fission process [58, 61, 82, 93].

Fission of the annular gap junction vesicle poses a topological challenge in that it has a double membrane, and unlike the thin neck of the gap junction bud, it has a larger area that has to be constricted to achieve vesicle fission. Similar challenges are faced by mitochondria, which can be as large as or larger than annular gap junction vesicles. Mitochondria, like annular gap junction vesicles, are double membrane-bound organelles. During the mitochondrial fission process, two members of the dynamin superfamily, dynamin-related proteins (Dnm1) in yeast, and dynamin-related protein (DRP1) in mammals, have been demonstrated to assemble into punctate structures on mitochondria surfaces [168–170]. These molecules facilitate the constriction and subsequent scissoring of the mitochondrium into two smaller mitochondria [169, 171, 172]. A similar process is suggested for the annular gap junction vesicle fission process. The current data is consistent with a role for dynamin in the scissoring needed to release annular gap junction vesicles into the cytoplasm as well as fission of the annular gap junction vesicle. Such fissions may be critical to gap junction protein degradation or recycling.

### Annular gap junction degradation and potential recycling

Cells have developed three principal degradation pathways: the proteasomal, the endo-lysosomal, and the phago-/lysosomal system (termed macroautophagy or simply autophagy) and all three serve specific cellular functions. The two latter degradation systems utilize the lysosome for final degradation. All three degradation systems have been implicated previously at various steps in the regulation of gap junction stability and connexin degradation [19, 173–178]. Since annular gap junction vesicles are highly oligomeric multi-subunit protein assemblies, their degradation by the proteasome (that is designed to degrade unfolded polypeptides that need to be inserted into the tubular core of the cytoplasmically located proteasome) appears unlikely, and to our knowledge, no evidence exists that would suggest proteasome-mediated degradation of assembled gap junction plaques, or of annular gap junction vesicles. Yet, inhibiting the proteasome can increase the number of gap junction channels and plaques in the plasma membrane [179, 180]. However this increase in gap junctions is most likely indirect due to decreasing degradation of connexin polypeptides shortly after their biosynthesis [10]. Both autophagosomal as well as endo-lysosomal degradation of annular gap junctions has been described and several recent reviews address annular gap junction degradation in great detail [92, 98, 121, 181–183]. We thus only briefly discuss this topic.

### Autophagosomal degradation

Several recent studies report the degradation of annular gap junctions by autophagy in situ as well as in cells in culture [16, 18–20, 158] (Fig. 8, bottom right). These studies correlate with older ultra-structural analysis in tissues [174, 177, 184, 185]. Autophagy degrades protein aggregates, organelles and other structures that are already present in the cytoplasm, under both starvation and un-starved conditions [92, 124, 186]. The structural characteristics of annular gap junctions together with the fact that autophagy serves as the default degradation pathway for cytoplasmically localized organelles and protein-aggregates suggests autophagy as the most logical cellular pathway for annular gap junction degradation. Multiple stages characteristic of autophagosome formation and maturation progressively encircling annular gap junction vesicles that coalesced into phagophores and fused with lysosomes have been detected to degrade annular gap junction vesicles in several independent ultrastructural analyses [18–20, 174, 177].

In addition, a large number of proteins essential or relevant for autophagosome formation and autophagic degradation (termed Atg-proteins) have been characterized and can be used as reliable markers for autophagic degradation. Of these, the ubiquitin-like proteins Atg12 (that is conjugated to Atg5) and LC3/Atg8, Atg7, and the PI3-kinase complex-component Beclin-1 (BECN1)/Atg6 (essential for phagophore nucleation) are essential for autophagosome formation and autophagic degradation. All of them have been targeted and found important for annular gap junction degradation [16, 18–20], further supporting autophagic degradation of annular gap junctions.

The ubiquitin-binding protein, p62 or sequestosome 1 (p62/SQSTM1) was found to colocalize with gap junction plaques in HeLa, COS7, and primary pulmonary artery endothelial cells [16, 18, 20]. p62/SQSTM1 recognizes and interacts via its UBA-domain with poly-ubiquitinated proteins [187–189] and delivers poly-ubiquitinated (Lys63-linked) oligomeric protein complexes to the autophagic degradation pathway [190, 191]. Depleting cells of p62 by RNAi knockdown significantly increased the number of annular gap junction vesicles [18, 20] further supporting the concept that p62 targets annular gap junction vesicles to autophagic degradation.

### Endo-lysosomal degradation

The endo-lysosomal system specifically degrades protein cargo that has been taken up at the plasma membrane in vesicles that mature into, or fuse with endosomes; or cargo that in other ways has entered endosomes. Membrane vesicles containing intact endocytosed gap junctions, or gap junctions apparently in the process of degradation have been observed in several ultrastructural studies [48, 151, 192–194], suggesting that annular gap junctions may fuse with lysosomes [151, 193, 194]. Also, the association of annular gap junctions with lysosomes, and the presence of acid phosphatase activity in annular gap junctions [48] further suggests that lysosomal degradation of annular gap junctions occurs following junctional internalization [33, 48, 49, 178]. More recently Leithe and colleagues [22] reported that upon TPA (a structural analog of the secondary messenger molecule diacylglycerol [DAG]) treatment of cells, internalized gap junctions are degraded by the endo-lysosomal pathway (Fig. 8, bottom left). The Leithe lab identified the protein Smurf2 (the HECT E3 ubiquitin ligase smad ubiqitination regulatory factor-2) as a critical factor that directs internalized gap junctions to the endo-lysosomal degradation pathway in TPA-treated cells [136].

Implicit in the degradation of annular gap junctions through the endo-lysosomal pathway is the fusion of a double-membrane vesicle containing densely packed gap junction channels (an annular gap junction vesicle) with a single-membrane endosome. The mechanism by which this is achieved has not been elucidated. In several reports it has been suggested that the inner membrane of the annular gap junction splits away from the outer membrane, generating a single-membraned cytoplasmic annular hemi-gap junction vesicle (with the inner annular gap junction membrane remaining inside) and thus could fuse with the single-membrane endosome [22, 195–197]. However, the signals that would drive such a gap junction channel splitting of annular gap junctions shortly after internalization are not understood. It is possible that the small membrane separations devoid of gap junction channels that have been observed by ultra-structural studies in annular gap junctions [61, 92, 184] (schematically depicted in Figs. 5, 8 and 10), likely corresponding to the “neck” of the plasma membrane invagination before forming the annular gap junction aid the fusion of annular gap junction vesicles with endosomes as the annular gap junction membranes are separated within these regions [61, 92]. The outer membrane of this region could fuse with the single-membrane endosome generating a larger vesicle (part endosome on one side, part gap junction plaque on the other side). The inner layer of the gap junction could then split away from the outer layer (for example based on increasing acidification as is typical for cargo/-receptor separation), forming an internal vesicle (complementing the inner hemi-gap junction plaque) that then could be degraded via the fusion with a lysosome. Analysis of cells incubated in media containing fluorescently labeled wheat germ agglutinin (WGA), a plasma membrane impermeant lectin that binds sialic acid and N-acetylglucosamine demonstrated that about 50 % of annular gap junctions (probably the ones that were internalized during the WGA-labeling period) contained a punctum of fluorescently labeled WGA [92]. Sialic acid and N-acetylglucosamine are carbohydrate moieties that are common on the surface of glycosylated plasma membrane proteins. Since connexins are not glycosylated and are densely packed within gap junction plaques, this data lends support to the idea that the small membrane separation observed in annular gap junctions indeed could correspond to the membrane domains where fusion with endosomes would occur.

Interestingly, TPA is a known potent activator of protein kinase C (PKC), and to promote hyper-phosphorylation and hyper-ubiquitination of Cx43 [22, 23, 110]. Based on these results it is tempting to speculate that the level of connexin phosphorylation and/or ubiquitination might allow cells to regulate which pathway endo-lysosomal versus phago-/lysosomal, endocytosed annular gap junction vesicles are sequestered and processed; an intriguing hypothesis considering that autophagosomal and endo-lysosomal processing could render different annular gap junction processing products.

### Annular gap junction recycling

A recycling of annular gap junctions back to the plasma membrane has been recently suggested [82, 91, 158]. However the evidence supporting this hypothesis is stillquestionable. Gilleron and colleagues demonstrated that buds released from annular gap junctions were positive for two GTPase Rab family members, Rab 4 and Rab 11 [82]. They suggested based on the colocalization of Cx43 with these Rab proteins, which are known to facilitate protein recycling, that connexins may recycle back to the plasma membrane. In these studies the buds were observed with immunofluorescence to be near gap junction plaques. Yet, the actual fusion/return of Cx43/Rab positive buds to the gap junction plaques/the plasma membrane remains to be demonstrated.

Carette and colleagues have, based on time-lapse imaging and transmission electron microscopic data, suggested that an entire annular gap junction vesicle may return to the plasma membrane to reform a gap junction plaque [158]. In addition, annular gap junction vesicles were suggested to release single membrane vesicles that could fuse with the plasma membrane and potentially participate in gap junction plaque formation [158]. While the possibility of gap junction protein recycling is intriguing, it should be noted that much of their suggested evidence was based on TEM observations of single membrane vesicles seen near or attached to gap junction plaques. It is not possible to definitely demonstrate movement (either to or from the gap junction plaques) with the still images collected with this technique. The time-lapse data provide convincing evidence for the dynamic nature of gap junction plaques and the annular gap junction vesicles. However, additional studies are needed in which the cell borders can clearly be discerned and internalization as well as re-insertion of gap junction components can be monitored convincingly to confirm the suggestions that annular gap junction vesicle connexins may indeed recycle back to the plasma membrane and importantly at what rate.

In addition to the proposed recycling of annular gap junction vesicle connexins or their buds, VanSlyke and colleagues have reported that cytosolic stress, induced by heat shock (42 °C), which did not alter the endocytosis of biotinylated Cx43 but rather inhibited Cx43 degradation, increased intracellular levels of connexin proteins and increased the accumulation of connexins into gap junction plaques. Based on these results, they suggested that high levels of connexin within intracellular compartments following cytosolic stress may allow for increased opportunities for connexins to recycle back to the plasma membrane to participate in gap junction plaque formation [180]. Finally, Boassa and collegues using a tetracysteine tag and successive FlAsH and ReAsH labeling that allowed the discrimination between newer and older connexin polypeptides provided evidence for the recycling of Cx43 back to the cell surface and the formation of new gap junctions during cell division [91]. However, additional studies are needed to definitively demonstrate gap junction protein recycling and the role of annular gap junction processing.

## Conclusions

It is now well accepted that gap junction internalization, which results in annular gap junction vesicle formation, is a major cellular pathway that significantly contributes to gap junction turnover and that this process utilizes the clathrin-mediated endocytosis machinery. Mutations in gap junction connexins can cause a number of devastating human diseases including inherited nonsyndromic hearing loss, X-linked Charcot-Marie-Tooth neuropathy, congenital eye lens cataracts, cardiac diseases such as hypertrophy, ischemia, and heart failure, a number of acute skin disorders, as well as craniofacial bone and other developmental defects (recently reviewed in ref. [198–202]). It is thought that mis-regulated gap junction plaque internalization, degradation and stabilization on the plasma membrane, which all result in aberrant levels of GJIC, contributes to the disease phenotype. Indeed, it has been recently reported that altered, non-physiological degradation rates of different connexins may cause disease ([203, 204]; reviewed in [200, 205]). Such results lend important support to the hypothesis that alterations in gap junction turnover are directly related to the development of many diseases. Thus, it will be crucial to explore and decipher the turnover characteristics of connexion 43, and of other connexions and their disease-relevant mutants since mutations in most, if not in all connexins has been suggested to be related or cause diseased phenotypes [205]. This is of particular interest for connexin types that do not encode conserved, known, canonical AP-2 clathrin adaptor binding sites (such as Cx26, Cx31, Cx31.1, Cx40 and Cx46) (Fisher and Falk, unpublished), since it is not clear if gap junctions assembled from these connexins are capable of turning over on their own, and whether they also implement the clathrin endocytic machinery. Moreover, as gap junctions contribute to physical cell-cell adhesion, many patho-/physiological processes that involve cell migration and cell-cell separation (such as cell migration in development and wound healing, mitosis, apoptosis, leukocyte extravasation, ischemia, hemorrhage, edema, cancer metastasis and others) [92] require the removal of gap junctions from the plasma membrane, and mis-regulation of this process may further contribute to disease. This would be especially true during development when the need for migration, differentiation and cell-cell separation are critical.

Exploring annular gap junction degradation processes via autophagosomal versus endo-lysosomal mechanisms appears important as well, as degrading annular gap junctions by different cellular mechanisms bears the potential for different outcomes; an interesting hypothesis especially when considering a potential re-use of connexin polypeptides, gap junction hemi-channels, or even entire gap junction channels, rather than their degradation. Finally, as gap junction biosynthesis is a complex, time-consuming and energetically costly process, the recently suggested concept that internalized gap junctions could be re-used by re-inserting them into the plasma membrane [158] is a provocative, yet intriguing concept. Research in the coming years promises to answer at least some of these exciting open questions.

## Abbreviations

AGJ: annular gap junction
AKT: kinase Protein kinase B (PKB)
AP-2: clathrin adaptor protein complex 2
CLASPs: clathrin associated sorting proteins
CME: clathrin mediated endocytosis
Cx: Connexin
Cx43-CT: carboxy-terminal domain of connexin 43
Dab2: disabled 2
DAG: diacylglycerol
E1: Ub-activating
E2: Ub-conjugating
E3: Ub-ligase enzyme
EGF: epidermal growth factor
Eps 15: epidermal growth factor receptor substrate 15
GFP: green fluorescent protein
GJ: gap junction
GJIC: gap junction intercellular communication
GTPase: enzyme that binds to guanosine triphosphate
K63-poly-Ub: a protein modification that mediates protein endocytosis and degradation
LAMP: lysosomal associated membrane protein
LC3: microtubule-associated protein light chain 3
MAGUK: membrane-associated guanylate kinase-family member
MAPK: mitogen-activated protein kinase
Nedd4: neuronal precursor cell-expressed developmentally down-regulated 4
PDZ-2: amino acid structural domain with the following three proteins: P (post synaptic density protein), D (*Drosophila* disc large tumor suppressor) & Z (Zonula occludens-1 protein)
PKA: protein kinase A
PKC: protein kinase C
Q-dot: quantum dot
RNAi: RNA interference
S1, S2, S3: connexin 43 adaptor-protein 2 (AP-2) binding sites (canonical tyrosine- based sorting signals of the type ‘YXXΦ’ where Φ is a bulky hydrophobic amino acid)
Smurf2: SMAD ubiquitination regulatory factor 2
SQSTM1: sequestosome 1
TEM: transmission electron microscopy
TPA: 12-O-tetradecanoylphorbol 13-acetate
Trim21: tripartite motif-containing protein 21
Ub: ubiquitin
VEGF: vascular endothelial growth factor
Wwp1: WW domain containing E3 ubiquitin protein ligase 1
ZO-1: zonula occludens-1 protein
